# Survival outcome prediction of esophageal squamous cell carcinoma patients based on radiomics and mutation signature

**DOI:** 10.1186/s40644-024-00821-5

**Published:** 2025-01-31

**Authors:** Ting Yan, Zhenpeng Yan, Guohui Chen, Songrui Xu, Chenxuan Wu, Qichao Zhou, Guolan Wang, Ying Li, Mengjiu Jia, Xiaofei Zhuang, Jie Yang, Lili Liu, Lu Wang, Qinglu Wu, Bin Wang, Tianyi Yan

**Affiliations:** 1 Second Clinical Medical College, Shanxi Medical University, Taiyuan, Shanxi 030001 People’s Republic of China; 2 Translational Medicine Research Center, Shanxi Medical University, Taiyuan, Shanxi 030001 People’s Republic of China; 3 School of Life Science, Beijing Institute of Technology, Beijing, People’s Republic of China; 4 School of Computer Information Engineering, Shanxi Technology and Business University, Taiyuan, Shanxi 030006 People’s Republic of China; 5 College of Information and Computer, Taiyuan University of Technology, Taiyuan, Shanxi 030024 People’s Republic of China; 6 Department of Thoracic Surgery, Shanxi Cancer Hospital, Taiyuan, Shanxi 030013 People’s Republic of China; 7 Department of Gastroenterology, Second Hospital of Shanxi Medical University, Taiyuan, Shanxi 030001 People’s Republic of China

**Keywords:** Esophageal squamous cell carcinoma, Mutation signature, Nomogram, Prognosis, Radiomics

## Abstract

**Background:**

The present study aimed to develop a nomogram model for predicting overall survival (OS) in esophageal squamous cell carcinoma (ESCC) patients.

**Methods:**

A total of 205 patients with ESCC were enrolled and randomly divided into a training cohort (*n* = 153) and a test cohort (*n* = 52) at a ratio of 7:3. Multivariate Cox regression was used to construct the radiomics model based on CT data. The mutation signature was constructed based on whole genome sequencing data and found to be significantly associated with the prognosis of patients with ESCC. A nomogram model combining the Rad-score and mutation signature was constructed. An integrated nomogram model combining the Rad-score, mutation signature, and clinical factors was constructed.

**Results:**

A total of 8 CT features were selected for multivariate Cox regression analysis to determine whether the Rad-score was significantly correlated with OS. The area under the curve (AUC) of the radiomics model was 0.834 (95% CI, 0.767–0.900) for the training cohort and 0.733 (95% CI, 0.574–0.892) for the test cohort. The Rad-score, S3, and S6 were used to construct an integrated RM nomogram. The predictive performance of the RM nomogram model was better than that of the radiomics model, with an AUC of 0. 830 (95% CI, 0.761–0.899) in the training cohort and 0.793 (95% CI, 0.653–0.934) in the test cohort. The Rad-score, TNM stage, lymph node metastasis status, S3, and S6 were used to construct an integrated RMC nomogram. The predictive performance of the RMC nomogram model was better than that of the radiomics model and RM nomogram model, with an AUC of 0. 862 (95% CI, 0.795–0.928) in the training cohort and 0. 837 (95% CI, 0.705–0.969) in the test cohort.

**Conclusion:**

An integrated nomogram model combining the Rad-score, mutation signature, and clinical factors can better predict the prognosis of patients with ESCC.

**Supplementary Information:**

The online version contains supplementary material available at 10.1186/s40644-024-00821-5.

## Background

Esophageal cancer (EC) is a leading cause of cancer death worldwide, ranking seventh in incidence and sixth in overall mortality [1]. Esophageal squamous cell carcinomas (ESCC) occur in 90% of patients with esophageal cancer [2]. After surgical treatment, the prognosis for patients with locally advanced EC remains poor, with a 5-year survival rate of only 25% [3]. For patients with early-stage disease, surgery, usually in combination with neoadjuvant therapy or chemoradiotherapy, offers the best chance of curative treatment [4, 5]. However, despite improvements in patient care, overall survival (OS) rates remain low [6]. Hence, effective means to preoperatively predict the prognosis of ESCC patients are necessary.

Radiomics refers to the extraction of a large number of high-dimensional quantitative features from multimodal medical images such as computed tomography (CT), magnetic resonance imaging (MRI), positron emission tomography (PET), and ultrasound (US) [7]. Radiomics features capture tissue and lesion characteristics, such as heterogeneity and shape, and can be used alone or in combination with demographic, histological, genomic, or proteomic data for clinical problem solving [8]. Numerous studies have constructed radiomics-based models that contribute to the prediction of cancer metastasis, diagnosis, prognosis, and treatment response [9]. For example, the radiomics model based on liver portal venous phase CT can better predict lymph node metastasis in biliary tract cancer patients [10]; The radiomics features of contrast-enhanced CT can help to predict tumor recurrence in early hepatocellular carcinoma patients [11]; The MRI-based radiomics feature model can predict the disease-free survival and overall survival of patients with ESCC [12].

Significant information is contained in the data of somatic mutations, which are present in all cells of the human body and occur throughout life. They are the consequence of multiple mutational processes, including the intrinsic slight infidelity of the DNA replication machinery, exogenous or endogenous mutagen exposures, enzymatic modification of DNA and defective DNA repair. Different mutational processes generate unique combinations of mutation types, termed “Mutational Signatures” (https://cancer.sanger.ac.uk/signatures/). Mutation signatures can not only identify cancer etiologies and the causes of driver mutations but also have both prognostic and therapeutic significance in cancer [13]. For example, integrating the mutation signatures of all classes can predict cancer patients with BRCA1 or BRCA2 deficiency [14]; APOBEC-associated SNV signatures are associated with the sensitivity of breast, ovarian, and other cancer cell lines to ATR inhibition [15]; The COSMIC signature 18 is enriched in neuroblastoma [16], colon cancer [17], pediatric leukemia [18], and rhabdomyosarcoma [19] and may benefit from therapies that exploit ROS; and the COSMIC signature 17b may induce KRAS/NRAS and EGFR driving mutations, leading to cetuximab resistance in colorectal cancer patients [20], resulting in poor progression-free survival in colorectal cancer patients [21]. Therefore, mutation signatures may be helpful in predicting the prognosis of patients with ESCC.

In the present study, we constructed a nomogram model based on radiomics, mutation signatures, and clinical factors to evaluate the prognosis of patients with ESCC. In addition, we attempted to reveal the biological significance of radiomics features.

## Materials and methods

### Patients and clinical characteristics

The entire dataset was obtained from the Institutional Picture Archiving and Communication System (PACS) at Shanxi Cancer Hospital from February 2016 to October 2018. This study was approved by the ethical committees of Shanxi Medical University. The inclusion criteria were as follows: (1) had pathologically confirmed ESCC; (2) underwent surgery for ESCC; (3) had standard contrast-enhanced CT performed preoperatively; (4) had whole-genome sequence analysis performed in ‘508 cohort’, which was investigated in 2020 [22]; and (5) had complete clinical and follow-up information available.

Clinical characteristics including age, gender, tumor location (upper, middle, lower), drinking history, smoking history, genetic alterations, and pathologic features, such as depth of invasion, TNM stage, and lymph node metastasis information were collected from patient records. These clinicopathologic characteristics are presented in Table 1.

**Table 1:** Patient and tumor characteristics in the training and test cohorts

	Training(*N* = 153)	Test (*N* = 52)	*P* Value
Gender			0.125
Male	109(71.2%)	31(59.6%)	
Female	44(28.8%)	21(40.4%)	
Age			0.483
Median (IQR)	60(54, 66)	60(57, 65)	
Location			0.226
Up	6(3.9%)	2(3.9%)	
Mid	99(64.7%)	40(76.9%)	
Down	48(31.4%)	10(19.2%)	
Drinking			0.103
Yes	64(41.8%)	15(28.8%)	
No	89(58.2%)	37(71.2%)	
Smoking			0.331
Yes	90(58.8%)	26(50.0%)	
No	63(41.2%)	26(50.0%)	
Genetic History			0.575
Yes	35(22.9%)	14(26.9%)	
No	118(77.1%)	38(73.1%)	
Grade			0.123
G1	11(7.2%)	0(0.0%)	
G2	112(73.2%)	40(76.9%)	
G3	30(19.6%)	12(23.1%)	
TNM			0.369
I	16(10.5%)	9(17.3%)	
II	80(52.3%)	27(51.9%)	
III	57(37.2%)	16(30.8%)	
Lymph Node Metastasis			0.195
Yes	70(45.8%)	18(34.6%)	
No	83(54.2%)	34(65.4%)	

All patients were followed up every 1–3 months during the first 2 years, every 6 months during years 2–5, and annually thereafter. Overall survival (OS) was defined as the interval between treatment initiation and the occurrence of death. Patients were censored at the time of withdrawal from treatment, the last follow-up, or the study end date.

### Acquire and segment the CT images

All patients underwent contrast-enhanced CT using a 64-channel multidetector CT scanner (LightSpeed VCT, GE Medical Systems, Milwaukee, Wis, USA). The acquisition parameters were as follows: 120 kV; 160 mA; 0.5-s rotation time; detector collimation: 64 × 0.625 mm; field of view: 350 mm × 350 mm; and matrix: 512 × 512. After routine nonenhanced CT, contrast-enhanced CT was performed after a 25-s delay following intravenous administration of 85 mL of iodinated contrast material (Ultravist 370; Bayer Schering Pharma, Berlin, Germany) at a rate of 3.0 mL/s with a pump injector (Ulrich CT Plus 150, Ulrich Medical, Ulm, Germany). All the images were reconstructed with a thick 5.0 mm slice. For feature selection, we converted the image format from DICOM to NII without applying any preprocessing.

**Fig. 1:**
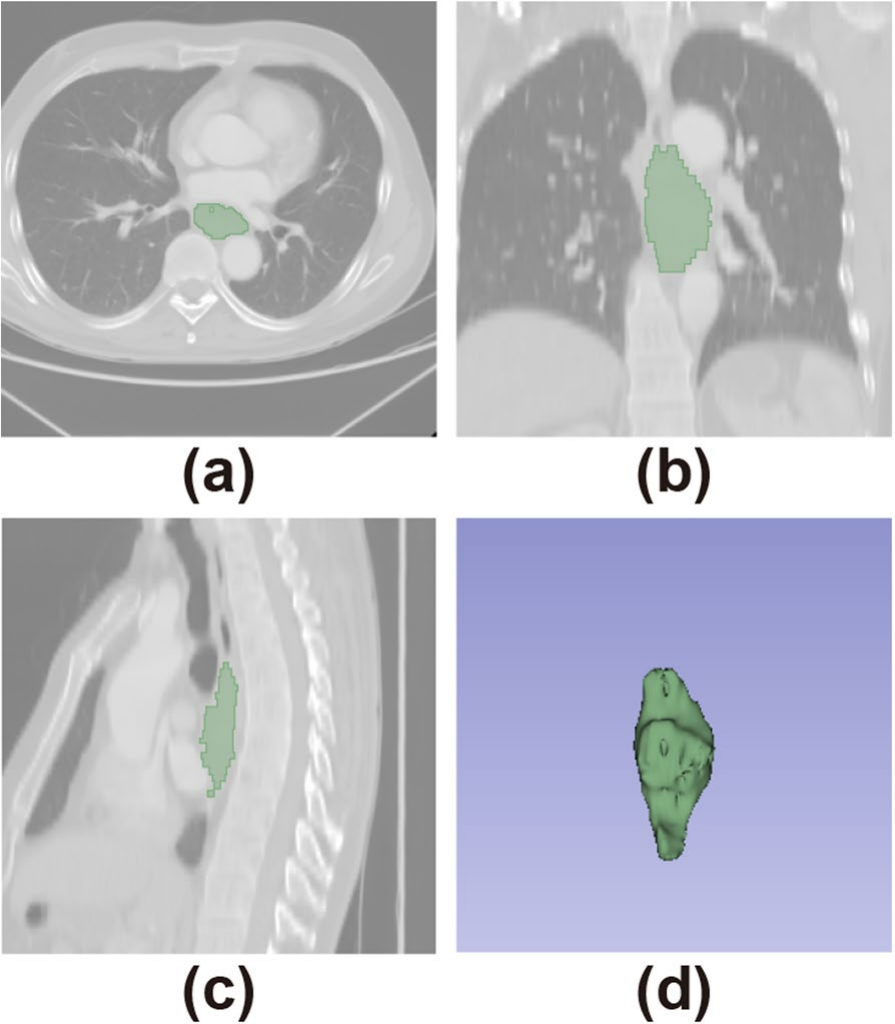
Sample ROI for CT images. **a** Segmentation on horizontal slices. **b** Segmentation on the coronal section. **c** Segmentation on sagittal slice. **d** 3D image of ROI

We performed three-dimensional manual segmentation by using 3D-Slicer software (https://www.slicer.org), which is an open platform for medical image processing. The chief physician of Shanxi Cancer Hospital, who has more than five years of experience in interpreting chest radiology, outlined the tumor regions for each CT image layer, and the tumor segmentation was guided and verified by a specialist. For each CT image, the region of interest (ROI) included the necrotic and bleeding areas within the lesion, when the esophageal wall showed focal thickening of more than 5 mm on transverse imaging, the esophageal wall was regarded as abnormal and included in the ROI, the segmentation example is shown in Fig. 1.

### Extract the radiomics features

We performed the calculations through our custom Python scripts (Python 3.7, https://www.python.org) for radiomics feature extraction based on the segmentation results. A total of 842 features were obtained by calling feature calculations in the pyradiomics package (open-source python package; https://pyradiomics.readthedocs.io/en/latest/), which included the following 4 categories: (1) first-order statistical features; (2) size- and shape-based features; (3) texture features; and (4) wavelet features; and 5 typical matrixes: gray-level co-occurrence matrix (GLCM), gray-Level run length matrix (GLRLM), gray-level size zone matrix (GLSZM), gray-level dependence matrix (GLDM) and neighboring gray-tone difference matrix (NGTDM).

### Select the radiomics features and construct the Rad-score

First, we performed Z score normalization for the quantitative features in the training and test cohorts. After data normalization, features were screened by the Spearman correlation coefficient, and only one feature was retained among the features with a high correlation (correlation coefficient > 0.95). Additionally, univariate Cox regression analysis was used to evaluate the correlation between radiomics features and OS in the training cohort. Features with *p* values < 0.05 were included in the LASSO regression analysis alone, and tenfold cross-validation was performed to further screen for features. Multivariate Cox regression analysis was subsequently used to construct the prediction model, and stepwise regression analysis was used to select the best model. Finally, radiomics features and radiomics score (Rad-score) were calculated from the features and their regression coefficients (β). The β is the weighted correlation coefficient in multivariate Cox regression analysis. An ROC curve was used to evaluate the prognostic accuracy of the Rad-score. All patients were divided into high-risk and low-risk groups according to the median cutoff value of the Rad-score. The association between the Rad-score and the prognosis of ESCC patients was evaluated by Kaplan–Meier survival analysis in the training cohort and test cohort.

### Analysis of the mutation signatures

We performed whole-genome sequencing (WGS) on ESCC tumors and matched paracancerous esophageal tissues [22]. The contributions of different mutation signatures were identified for each sample according to the distribution of the six substitution classes (C > A, C > G, C > T, T > A, T > C, and T > G), and the bases immediately 5´ and 3´ to the mutated base, producing 96 possible mutation subtypes, which were compared to the known 79 COSMIC signatures. The proportion of mutational signatures in the sample was assessed, the truncation value was generated by the R package 'cutoff', and the sample was divided into high and low groups. Subsequently, the association between the mutation signature and the prognosis of ESCC patients was evaluated by Kaplan–Meier survival analysis.

### Construct and evaluate the nomogram

First, multivariate Cox regression analysis was used to establish an RM nomogram model for the training cohort by combining the Rad-score with the prognostic mutation signature in ESCC patients. Then, an RMC nomogram model of the training cohort was constructed by combining the Rad-score with the mutation signature and clinical factors with the prognosis of patients with ESCC. The ROC curve was used to evaluate the prognostic accuracy of the RM nomogram model and RMC nomogram model. Decision curve analysis (DCA) was performed to analyze the clinical usefulness of the RMC nomogram by quantitatively measuring the net benefit at different threshold probabilities.

### Statistical analysis

All the statistical analyses were performed using Python (version 3.7) software and software (version 4.2.2). The following R packages were used: the glmnet package was used for LASSO regression model analysis, the pROC package was used for receiver operating characteristic (ROC) curve analysis, the survival package was used for the Kaplan–Meier survival analysis, the SomaticSignatures and maftools package was used for mutation signature analysis, and the rms package was used for nomogram construction and calibration.

## Results

### Clinical characteristics of patients

**Fig. 2:**
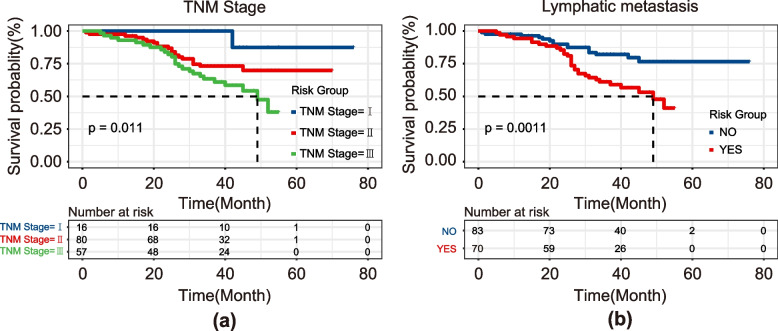
Kaplan–Meier curve for the TNM stage (**a**) and lymph node metastasis (**b**) groups

A total of 205 patients (140 men, 65 women) with a mean age of 60.3 years ± 7.7 years were included. Stratified random sampling was used and the patients were divided into two groups, the training cohort (109 men, 44 women) and the test cohort (31 men, 21 women), at a ratio of 7:3. The clinical characteristics and statistics of the training and test cohorts are summarized in Table 1. There were no significant differences in age, gender, tumor location, drinking history, smoking history, genetic alterations, depth of invasion, TNM stage, or lymph node metastasis between the training and test cohorts (*p* > 0.05), which justified their use as training and test cohorts. Moreover, according to the Kaplan–Meier curve shown in Fig. 2, the patients were divided into three groups according to TNM stage, and the group with a higher TNM stage had a worse prognosis. The log-rank test showed that *p* < 0.05 was significantly related to the survival of the three groups. According to lymph node metastasis, the patients were divided into a metastasis group and non-metastasis group, and the metastasis group had a worse prognosis; moreover, the survival status of the two groups was significantly different according to the log-rank test. Therefore, the TNM stage and lymph node metastasis status are closely related to the prognosis of patients with ESCC.

### Selection of the radiomics features and construction of the Rad-score

**Fig. 3:**
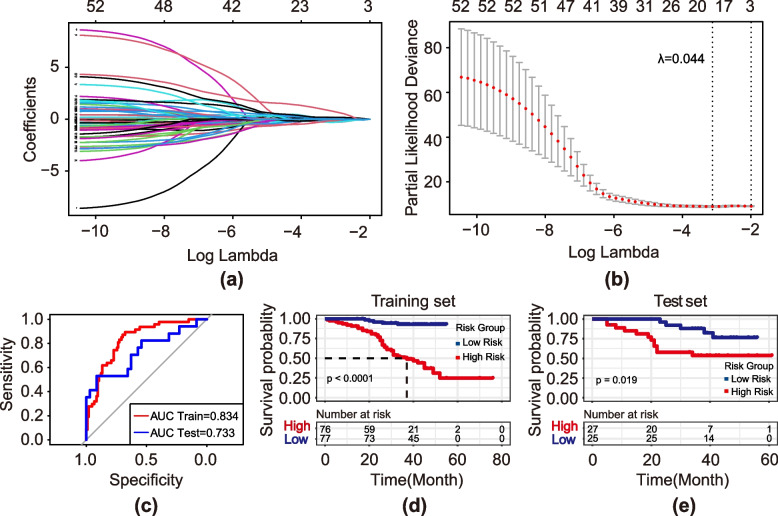
Construction of the radiomics model. **a** LASSO coefficient profiles of the 52 radiomics features. **b** Identification of the optimal penalization coefficient lambda (λ) in the LASSO model used tenfold cross-validation and the minimum criterion. As a result, a λ value of 0.044 was selected. **c** The ROC curve was used to assess the discriminative performance of the radiomics signature for survival status. The ROC in the training cohort was 0.834 (95% CI: 0.767–0.900); the ROC in the test cohort was 0.733 (95% CI: 0.574–0.892). Kaplan–Meier curve of risk grouping in the training cohort **(d)** and test cohort **(e)**

A total of 842 features were extracted from the CT images. After screening by the Spearman correlation coefficient, 359 related features were obtained, and the pairwise correlation was less than 0.95 (Fig. S1a). According to univariate statistical tests (*p* < 0.05), we screened out 52 features. The method based on the LASSO regression algorithm was subsequently applied to the training cohort. With increasing lambda, the number of features gradually decreases. When lambda.min was 0.044, 17 features had nonzero coefficients (Fig. 3a and b). Finally, the 17 features were used to construct the multivariate Cox regression model, and stepwise regression analysis was adopted to screen for secondary features. The model ultimately included 8 features: first-order mean HHH, glcm idmn HHH, glcm cluster shade HLL, glcm correlation LHL, glrlm run entropy LLL, glszm large area high gray level emphasis LLL, glszm size zone nonuniformity normalized LLL and glszm gray level variance (Fig. S1b). The discriminative ability of the survival status based on the radiomics signatures was assessed by ROC analysis in two cohorts (Fig. 3c). The AUC of the radiomics model in the training cohort was 0.834 (95% CI, 0.767–0.900), and that in the test cohort was 0.733 (95% CI, 0.574–0.892).

The patients were separated into high-risk and low-risk groups based on the median cutoff value of the Rad-score. Kaplan–Meier analysis revealed that the Rad-score was significantly associated with the prognosis of patients with ESCC, and the log-rank test showed that there were significant differences in survival between the high-risk group and the low-risk group (Fig. 3d and e). We found that the greater the Rad-score was, the worse the patient's prognosis.

**Fig. 4:**
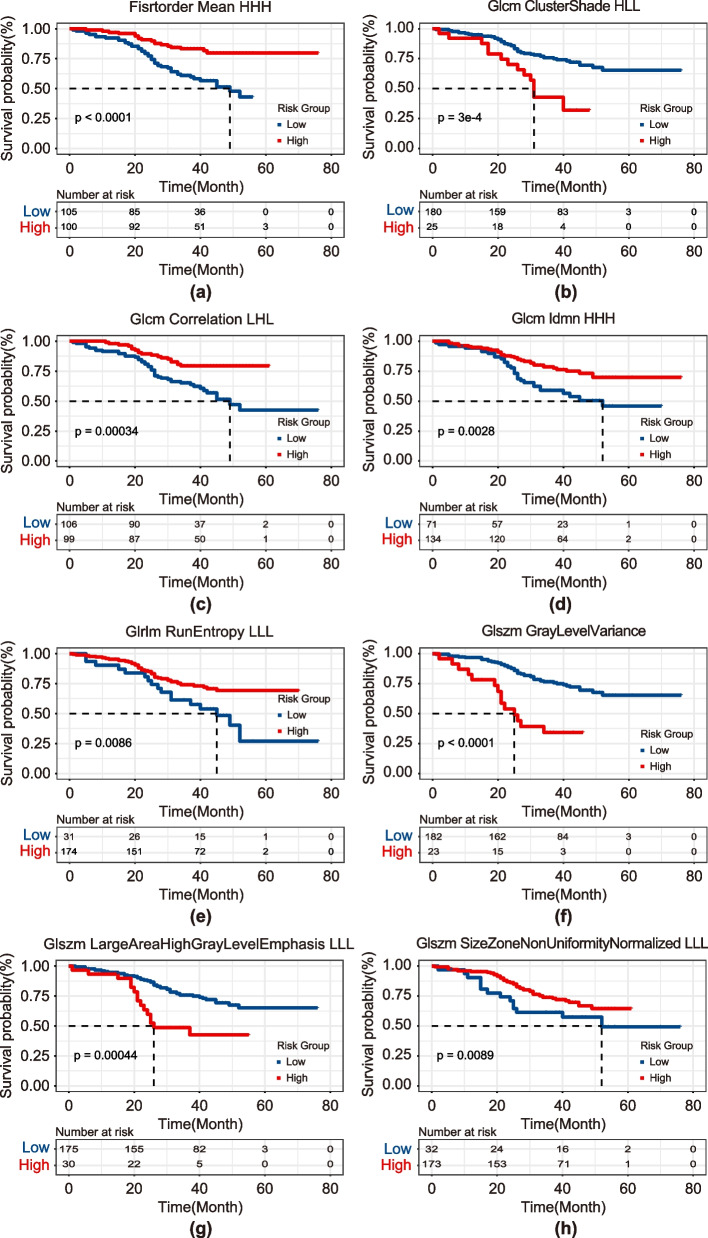
Kaplan–Meier curve of the 8 radiomics features

We independently assessed the impact of each radiomics feature on the prognosis of patients with ESCC and found that all of the features had prognostic significance (Fig. 4). We found that the greater the GLCM cluster shade, GLSZM large area high gray level emphasis, and GLSZM gray level variance, the worse the patient's prognosis was, and the opposite was true for other radiomics features.

### Mutation signatures for prognosis

**Fig. 5:**
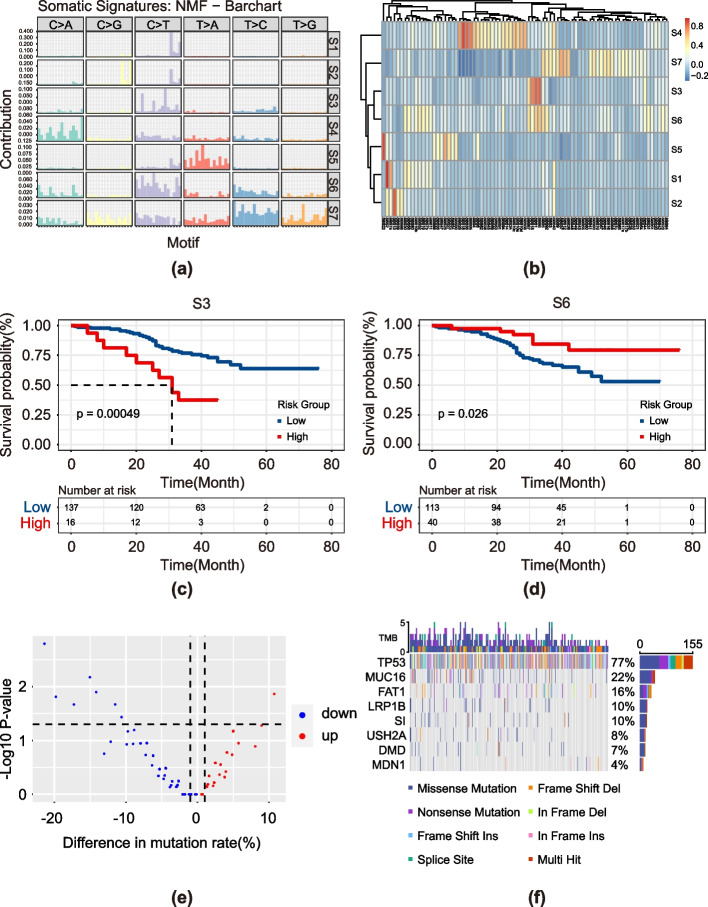
Mutational processes in ESCC. **a** Seven mutation signatures detected in ESCC (S1 − S7). **b** Cosine similarity between the 79 cosmic signature (horizontal axis) and ESCC cohort 7 signatures. Kaplan–Meier curve of the S3 **(c)** and S6 **(d)**. **e** Volcano plot indicating mutational rate differences (x-axis) for each gene (represented as dots), and significance (y-axis, negative-log scale). **f** Mutation status of 8 differentially mutated genes

To better understand the contribution of these mutations to ESCC etiology, we investigated mutational signatures. Using a modified nonnegative matrix factorization (NMF) algorithm we identified 7 mutational signatures (S1-S7) in the 205-WGS cohort (Fig. S2a and 5a). In addition to S6 and S7, all the other signatures corresponded to mutation signatures in the Catalogue of Somatic Mutations in Cancer (COSMIC) database (Fig. 5b). S1 and S2 were related to APOBEC (apolipoprotein B mRNA editing enzyme, catalytic polypeptide-like) activity, S3 was related to spontaneous deamination of 5-methyicytisine, S4 was related to damage by reactive oxygen species, and S5 was related to aristolochic acid exposure (Table 2). We then quantified the relevant contributions of the seven mutation signatures to each patient, with all but S5 contributing strongly (Fig. S2b).

**Table 2:** Relationships between the identified signature and COSMIC curated signatures

Signatures	COSMIC Signature	Related to
S1	COSMIC Signature 2	APOBEC
S2	COSMIC Signature 13	APOBEC
S3	COSMIC Signature 1	Spontaneous deamination of 5-methyicytisine
S4	COSMIC Signature 18	Damage by reactive oxygen species
S5	COSMIC Signature 22	Aristolochic acid exposure
S6	No similar COSMIC signatures	NA
S7	No similar COSMIC signatures	NA

We correlated the proportions of patients with different mutational signatures and OS. Kaplan–Meier analysis revealed that S3 and S6 were significantly associated with the prognosis of patients with ESCC (Fig. 5c and d, log-rank test, *p* < 0.05). We found that the greater the proportion of S3 macrophages was, the worse the patient's prognosis was, and the opposite was true for S6.

We determined the differences in the genomic landscape between patients with high and low proportions of S6 and identified eight differentially mutated genes, TP53, MUC16, FAT1, LRP18, SI, USH2A, DMD and MDN1 (Fig. 5e and f). We separately analyzed the proportion of S6 in patients with or without these eight mutated genes (Fig. S3). A greater proportion of patients with MND1 mutations had S6, and the difference in the proportion of S6 among the other genes was reversed, which was consistent with the difference analysis results.

### Construction and evaluation of the nomogram

**Fig. 6:**
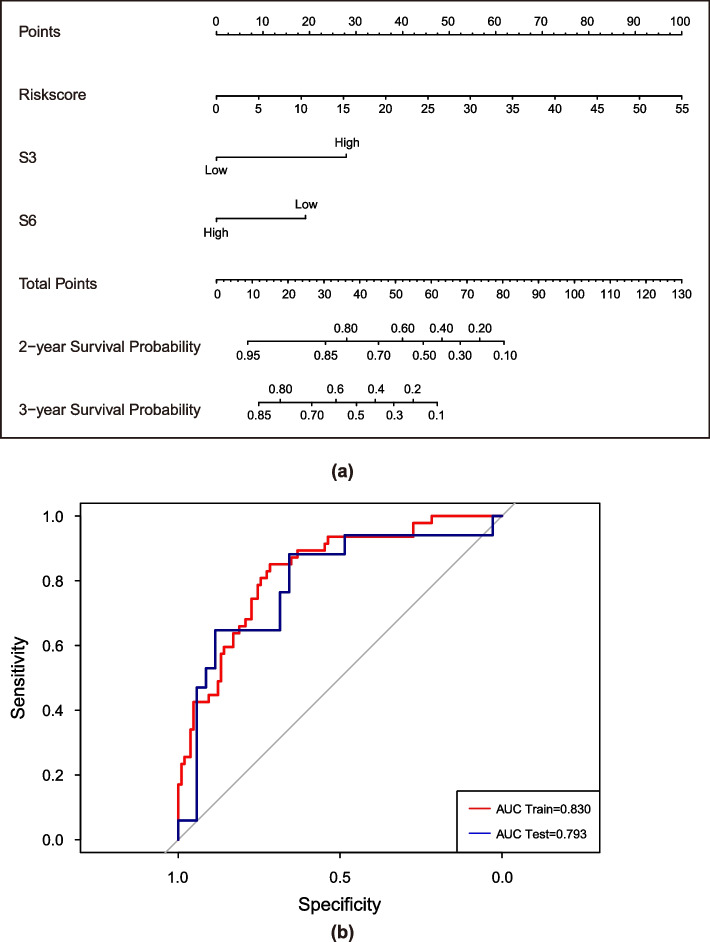
**a** The RM nomogram combining the Rad-score and mutation signature (S3 and S6). **b** ROCs of the training cohort and test cohort. The AUC of the RM nomogram model in the training cohort was 0.830 (95% CI, 0.761–0.899), and that in the test cohort was 0.793 (95% CI, 0.653–0.934)

The RM nomogram constructed by combining the Rad-score and mutation signature (S3 and S6) is shown in Fig. 6a. The AUC of the RM nomogram model in the training cohort was 0.830 (Fig. 6b, 95% CI, 0.761–0.899), and that in the test cohort was 0.793 (Fig. 6b, 95% CI, 0.653–0.934).

**Fig. 7:**
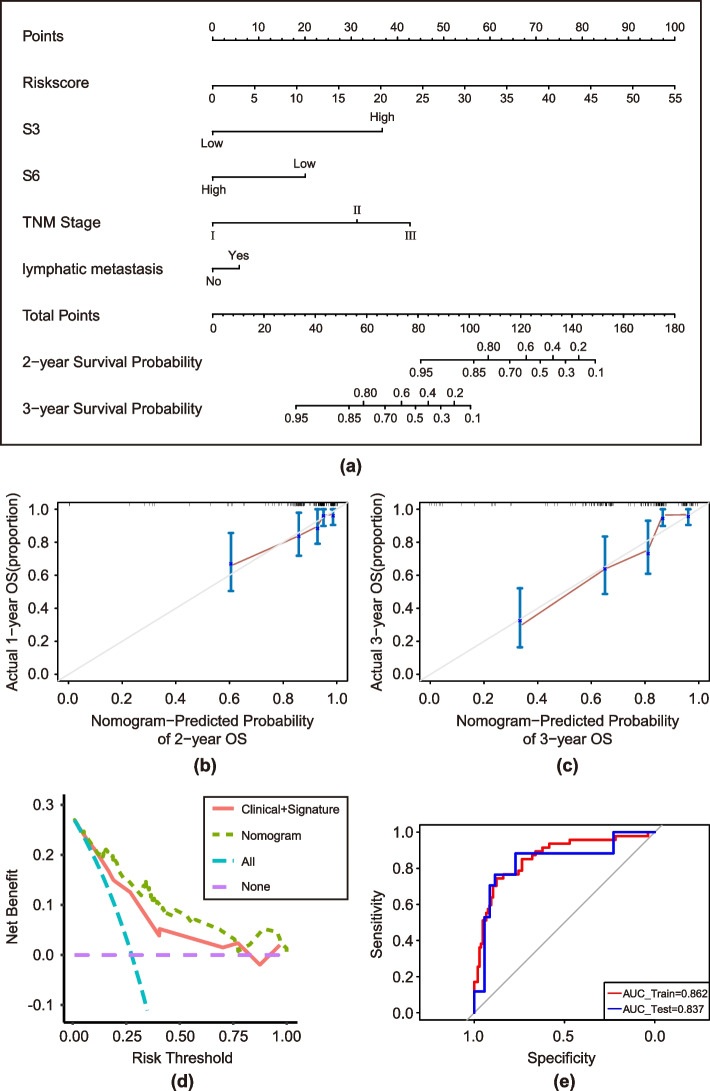
**a** The RMC nomogram combining the Rad-score, mutation signature (S3 and S6), and clinical factors (TNM stage and lymph node metastasis). **b** Calibration curve of the RMC nomogram for predicting 2-year OS. **c** Calibration curve of the RMC nomogram for predicting 3-year OS. **d** DCA of the RMC nomogram. The integrated nomogram model had better net benefits than did the traditional prediction model. **e** ROCs of the training cohort and test cohort. The AUC of the RMC nomogram model in the training cohort was 0.862 (95% CI, 0.795–0.928), and that in the test cohort was 0.837 (95% CI, 0.705–0.969)

The RMC nomogram constructed by combining the Rad-score, mutation signature (S3 and S6), and clinical factors (TNM stage and lymph node metastasis) is shown in Fig. 7a. The calibration curve of the nomogram showed a good agreement between the prediction and observation results (Fig. 7b and c). The DCA results showed that the predictive model combining the Rad-score, mutation signature, and clinical factors had a greater net benefit than the single-factor predictive model (Fig. 7d). The AUC of the RMC nomogram model in the training cohort was 0.862 (Fig. 7e, 95% CI, 0.795–0.928), and that in the test cohort was 0.837 (Fig. 7e, 95% CI, 0.705–0.969).

## Discussion

In this retrospective study, we established a CT-based radiomics model and an integrated nomogram model combining the Rad-score, mutation signature, and clinical factors to predict the prognosis of patients with ESCC. Compared with the single radiomics model, the integrated nomogram model has better predictive performance.

By predicting the prognosis of patients with ESCC, it is possible to determine the risk profile of patients with ESCC, thereby helping clinicians identify patients with poor prognoses who may be candidates for upgrading treatment and/or clinical trials. Conversely, if identified beforehand, patients with a good prognosis may have a favorable outcome with de-escalation therapy, which can avoid the physiological and economic toxicity of cancer treatment [23]. Features with diagnostic and prognostic potential may serve as a pre-operative biomarker to select ESCC patients who would benefit from adjuvant therapy [24]. The traditional prognosis is dependent on doctor observation, which differs greatly according to experience. Radiomics uses a large number of automatically extracted data representation algorithms to convert imaging data into high-dimensional minable feature spaces [25]. Perfusion analysis using computed tomography (CT) or magnetic resonance imaging (MRI), texture analysis, diffusion-weighted imaging (DWI), and positron emission tomography (PET) can be used to construct prognostic markers to improve outcomes in patients with esophageal cancer [25]. In tumor diagnosis, it can help distinguish between benign and malignant tumors [26–28], lymph node metastasis of tumors [29–31], or predict the prognosis of cancer patients [10, 32], and then make the correct diagnosis and treatment plan for patients [33]. Radiomics features can reflect pathological changes or physiological states in an organism. By analyzing the radiomics features, we can obtain information on tissue structure [34, 35], hemodynamics [36, 37], metabolic activity [38], etc., which is very important for the early diagnosis and treatment strategy decision. Radiomics features can also reveal the mechanism of disease initiation and progression, which shows great potential for personalized medical applications. Furthermore, the radiomics prediction model has the advantages of being noninvasive and economical and can guide the clinical treatment of cancer before surgery [39]. In this study, we analyzed all the acquired CT images and constructed a CT-based radiomics signature. The spearman correlation coefficient, univariate Cox, and LASSO-Cox methods were used to screen the radiomics features, and a multivariate Cox regression model was subsequently constructed to predict the prognosis of patients with ESCC. The results showed that the radiomics model had better predictive performance. The AUC of the training cohort was 0.834 (95% CI, 0.767–0.900), and that of the test cohort was 0.733 (95% CI, 0.574–0.892). We analyzed the radiomics features separately and found that they all had prognostic significance.

In the process of cancer development, the expression of several genes changes rapidly, resulting in the rapid proliferation and spread of cancer cells, and the formation of malignant tumors, ultimately complicating cancer occurrence and treatment [40–42]. Gene mutations are closely related to the occurrence of malignant tumors and are strongly related to the prognosis of cancer [42–46]. Mutation data contain much information; however, the relationship between gene mutations and cancer prognosis is mostly limited to a single gene [47–49]. The mutation signature was used to analyze the somatic mutations of the patients comprehensively. In addition to identifying cancer etiologies and the causes of driver mutations, analyzing mutational signatures can also lead to direct therapeutic and prognostic insights [13]. We constructed mutation signatures that are associated with the prognosis of patients with ESCC. The Rad-score and mutation signature were subsequently combined to construct an RM nomogram model, which exhibited better prediction performance compared than the Rad-score alone.

An important advantage of our study is that, when radiomics features were applied, not only were the radiomics features used to construct the Rad-score, but the prognostic significance of the radiomics features was also analyzed separately. This is because the individual analysis of radiomics features is necessary for the clinical application of radiomics and provides valuable information for predicting patient prognosis. Analysis of radiomics features alone is also helpful for exploring the biological significance of radiomics features.

Our study has a few limitations. First, this was a retrospective study, as a supplement to this study, more accurate data could be collected in combination with prospective studies to increase the robustness of the model. Second, the amount of data used in this study was small, and to better generalize the conclusions, we need to verify the results with additional datasets.

In conclusion, we developed an integrated nomogram model that combines the Rad-score, mutation signature, and clinical factors to predict the prognosis of patients with ESCC. In addition, we explored the biological significance of the radiomics features.

## Conclusions

We developed an integrated nomogram model combining the Rad-score, mutation signature, and clinical factors. The results showed that the integrated nomogram model could predict the prognosis of ESCC patients well.

## Supplementary Information

Supplementary Material 1. Fig. S1 (a) Spearman correlation between 359 features. The pairwise correlation was less than 0.95. (b) Forest plot of multivariate Cox regression analysis.

Supplementary Material 2. Fig. S2 (a) Upper, residual sum of squares (RSS) of different signature number selections. Lower, percentage of variance explained by the selection of different signature numbers. (b) Relative contributions of the seven mutational features.

Supplementary Material 3. Fig. S3 The proportion of S6 positive patients with or without these eight mutated genes.

## Data Availability

The data cohorts used and/or analyzed during the current study are available from the corresponding author on reasonable request.

## Abbreviations

OS: Overall Survival
EC: Esophageal Cancer
ESCC: Esophageal Squamous Cell Carcinoma
AUC: Area Under the Curve
CT: Computed Tomography
MRI: Magnetic Resonance Imaging
PET: Positron Emission Tomography
US: Ultrasound
PACS: Picture Archiving and Communication System
ROI: Region of Interest
GLCM: Gray-Level Cooccurrence Matrix
GLRLM: Gray Level Run Length Matrix
GLSZM: Gray Level Size Zone Matrix
GLDM: Gray Level Dependence Matrix
NGTDM: Neighboring Gray Tone Difference Matrix
Rad-score: Radiomics Score
WGS: Whole-Genome Sequencing
DCA: Decision Curve Analysis
NMF: Nonnegative Matrix Factorization
DWI: Diffusion-Weighted Imaging

## References

[1] Sung H, et al. Global Cancer Statistics 2020: GLOBOCAN estimates of incidence and mortality worldwide for 36 cancers in 185 countries. CA Cancer J Clin. 2021;71(3):209–49. 33538338 10.3322/caac.21660

[2] Yang H, et al. Long-term efficacy of neoadjuvant chemoradiotherapy plus surgery for the treatment of locally advanced esophageal squamous cell carcinoma: the NEOCRTEC5010 randomized clinical trial. JAMA Surg. 2021;156(8):721–9. 34160577 10.1001/jamasurg.2021.2373 PMC8223138

[3] Herskovic A, et al. Esophageal carcinoma advances in treatment results for locally advanced disease: review. Ann Oncol. 2012;23(5):1095–103. 22003242 10.1093/annonc/mdr433

[4] Allum WH, et al. Long-term results of a randomized trial of surgery with or without preoperative chemotherapy in esophageal cancer. J Clin Oncol. 2009;27(30):5062–7. 19770374 10.1200/JCO.2009.22.2083

[5] van Hagen P, et al. Preoperative chemoradiotherapy for esophageal or junctional cancer. N Engl J Med. 2012;366(22):2074–84. 22646630 10.1056/NEJMoa1112088

[6] Sah BR, et al. Radiomics in esophageal and gastric cancer. Abdom Radiol (NY). 2019;44(6):2048–58. 30116873 10.1007/s00261-018-1724-8 PMC6934409

[7] Liu Z, et al. The applications of radiomics in precision diagnosis and treatment of oncology: opportunities and challenges. Theranostics. 2019;9(5):1303–22. 30867832 10.7150/thno.30309 PMC6401507

[8] Mayerhoefer ME, et al. Introduction to radiomics. J Nucl Med. 2020;61(4):488–95. 32060219 10.2967/jnumed.118.222893 PMC9374044

[9] Zhang YP, et al. Artificial intelligence-driven radiomics study in cancer: the role of feature engineering and modeling. Mil Med Res. 2023;10(1):22. 37189155 10.1186/s40779-023-00458-8 PMC10186733

[10] Ji GW, et al. Biliary tract cancer at CT: a radiomics-based model to predict lymph node metastasis and survival outcomes. Radiology. 2019;290(1):90–8. 30325283 10.1148/radiol.2018181408

[11] Ji GW, et al. Radiomic features at contrast-enhanced CT predict recurrence in early stage hepatocellular carcinoma: a multi-institutional study. Radiology. 2020;294(3):568–79. 31934830 10.1148/radiol.2020191470

[12] Chu F, et al. Development and validation of MRI-based radiomics signatures models for prediction of disease-free survival and overall survival in patients with esophageal squamous cell carcinoma. Eur Radiol. 2022;32(9):5930–42. 35384460 10.1007/s00330-022-08776-6

[13] Brady SW, Gout AM, Zhang J. Therapeutic and prognostic insights from the analysis of cancer mutational signatures. Trends Genet. 2022;38(2):194–208. 34483003 10.1016/j.tig.2021.08.007 PMC8752466

[14] Davies H, et al. HRDetect is a predictor of BRCA1 and BRCA2 deficiency based on mutational signatures. Nat Med. 2017;23(4):517–25. 28288110 10.1038/nm.4292 PMC5833945

[15] Buisson R, et al. APOBEC3A and APOBEC3B activities render cancer cells susceptible to ATR inhibition. Cancer Res. 2017;77(17):4567–78. 28698210 10.1158/0008-5472.CAN-16-3389 PMC5609510

[16] Alexandrov LB, et al. Signatures of mutational processes in human cancer. Nature. 2013;500(7463):415–21. 23945592 10.1038/nature12477 PMC3776390

[17] Alexandrov LB, et al. The repertoire of mutational signatures in human cancer. Nature. 2020;578(7793):94–101. 32025018 10.1038/s41586-020-1943-3 PMC7054213

[18] McLeod C, et al. St. Jude Cloud: a pediatric cancer genomic data-sharing ecosystem. Cancer Discov. 2021;11(5):1082–99. 33408242 10.1158/2159-8290.CD-20-1230 PMC8102307

[19] Brady SW, et al. Pan-neuroblastoma analysis reveals age- and signature-associated driver alterations. Nat Commun. 2020;11(1):5183. 33056981 10.1038/s41467-020-18987-4 PMC7560655

[20] Misale S, et al. Emergence of KRAS mutations and acquired resistance to anti-EGFR therapy in colorectal cancer. Nature. 2012;486(7404):532–6. 22722830 10.1038/nature11156 PMC3927413

[21] Woolston A, et al. Mutational signatures impact the evolution of anti-EGFR antibody resistance in colorectal cancer. Nat Ecol Evol. 2021;5(7):1024–32. 34017094 10.1038/s41559-021-01470-8 PMC7611134

[22] Cui Y, et al. Whole-genome sequencing of 508 patients identifies key molecular features associated with poor prognosis in esophageal squamous cell carcinoma. Cell Res. 2020;30(10):902–13. 32398863 10.1038/s41422-020-0333-6 PMC7608103

[23] Bera K, et al. Predicting cancer outcomes with radiomics and artificial intelligence in radiology. Nat Rev Clin Oncol. 2022;19(2):132–46. 34663898 10.1038/s41571-021-00560-7 PMC9034765

[24] Li K, et al. A signature of saliva-derived exosomal small RNAs as predicting biomarker for esophageal carcinoma: a multicenter prospective study. Mol Cancer. 2022;21(1):21. 35042519 10.1186/s12943-022-01499-8 PMC8764835

[25] Hayano K, et al. Imaging biomarkers for the treatment of esophageal cancer. World J Gastroenterol. 2019;25(24):3021–9. 31293338 10.3748/wjg.v25.i24.3021 PMC6603816

[26] Zheng Y, et al. CT-based radiomics analysis of different machine learning models for differentiating benign and malignant parotid tumors. Eur Radiol. 2022;32(10):6953–64. 35484339 10.1007/s00330-022-08830-3

[27] Zhou J, et al. Diagnosis of benign and malignant breast lesions on DCE-MRI by using radiomics and deep learning with consideration of peritumor tissue. J Magn Reson Imaging. 2020;51(3):798–809. 31675151 10.1002/jmri.26981 PMC7709823

[28] Conti A, et al. Radiomics in breast cancer classification and prediction. Semin Cancer Biol. 2021;72:238–50. 32371013 10.1016/j.semcancer.2020.04.002

[29] Yu Y, et al. Magnetic resonance imaging radiomics predicts preoperative axillary lymph node metastasis to support surgical decisions and is associated with tumor microenvironment in invasive breast cancer: a machine learning, multicenter study. EBioMedicine. 2021;69:103460. 34233259 10.1016/j.ebiom.2021.103460 PMC8261009

[30] Yu Y, et al. Development and validation of a preoperative magnetic resonance imaging radiomics-based signature to predict axillary lymph node metastasis and disease-free survival in patients with early-stage breast cancer. JAMA Netw Open. 2020;3(12):e2028086. 33289845 10.1001/jamanetworkopen.2020.28086 PMC7724560

[31] Huang YQ, et al. Development and validation of a radiomics nomogram for preoperative prediction of lymph node metastasis in colorectal cancer. J Clin Oncol. 2016;34(18):2157–64. 27138577 10.1200/JCO.2015.65.9128

[32] Park H, et al. Radiomics signature on magnetic resonance imaging: association with disease-free survival in patients with invasive breast cancer. Clin Cancer Res. 2018;24(19):4705–14. 29914892 10.1158/1078-0432.CCR-17-3783

[33] Zhou S, et al. Deep radiomics-based fusion model for prediction of bevacizumab treatment response and outcome in patients with colorectal cancer liver metastases: a multicentre cohort study. EClinicalMedicine. 2023;65: 102271. 37869523 10.1016/j.eclinm.2023.102271 PMC10589780

[34] Oikonomou EK, et al. A novel machine learning-derived radiotranscriptomic signature of perivascular fat improves cardiac risk prediction using coronary CT angiography. Eur Heart J. 2019;40(43):3529–43. 31504423 10.1093/eurheartj/ehz592 PMC6855141

[35] Levi R, et al. CT-based radiomics can identify physiological modifications of bone structure related to subjects’ age and sex. Radiol Med. 2023;128(6):744–54. 37147473 10.1007/s11547-023-01641-6

[36] Yu L, et al. Radiomics features of pericoronary adipose tissue improve CT-FFR performance in predicting hemodynamically significant coronary artery stenosis. Eur Radiol. 2023;33(3):2004–14. 36258046 10.1007/s00330-022-09175-7

[37] Zhou K, et al. Incremental diagnostic value of radiomics signature of pericoronary adipose tissue for detecting functional myocardial ischemia: a multicenter study. Eur Radiol. 2023;33(5):3007–19. 36729175 10.1007/s00330-022-09377-z

[38] Lasorsa F, et al. Emerging hallmarks of metabolic reprogramming in prostate cancer. Int J Mol Sci. 2023;24(2):910. 36674430 10.3390/ijms24020910 PMC9863674

[39] Li G, et al. An MRI radiomics approach to predict survival and tumour-infiltrating macrophages in gliomas. Brain. 2022;145(3):1151–61. 35136934 10.1093/brain/awab340 PMC9050568

[40] Dietlein F, et al. Genome-wide analysis of somatic noncoding mutation patterns in cancer. Science. 2022;376(6589):eabg5601. 35389777 10.1126/science.abg5601 PMC9092060

[41] Stratton MR, Campbell PJ, Futreal PA. The cancer genome. Nature. 2009;458(7239):719–24. 19360079 10.1038/nature07943 PMC2821689

[42] Stephens PJ, et al. The landscape of cancer genes and mutational processes in breast cancer. Nature. 2012;486(7403):400–4. 22722201 10.1038/nature11017 PMC3428862

[43] Sabapathy K, Lane DP. Therapeutic targeting of p53: all mutants are equal, but some mutants are more equal than others. Nat Rev Clin Oncol. 2018;15(1):13–30. 28948977 10.1038/nrclinonc.2017.151

[44] Wardell CP, et al. Genomic characterization of biliary tract cancers identifies driver genes and predisposing mutations. J Hepatol. 2018;68(5):959–69. 29360550 10.1016/j.jhep.2018.01.009

[45] Walker BA, et al. Identification of novel mutational drivers reveals oncogene dependencies in multiple myeloma. Blood. 2018;132(6):587–97. 29884741 10.1182/blood-2018-03-840132 PMC6097138

[46] Hassan AB, Paraskeva C. Colorectal cancer prognosis: is it all mutation, mutation, mutation? Gut. 2005;54(9):1209–11. 16099785 10.1136/gut.2005.070946 PMC1774661

[47] Castellanos E, Feld E, Horn L. Driven by mutations: the predictive value of mutation subtype in EGFR-Mutated non-small cell lung cancer. J Thorac Oncol. 2017;12(4):612–23. 28017789 10.1016/j.jtho.2016.12.014

[48] Li X, et al. Association of MUC16 mutation with tumor mutation load and outcomes in patients with gastric cancer. JAMA Oncol. 2018;4(12):1691–8. 30098163 10.1001/jamaoncol.2018.2805 PMC6440715

[49] Momeni-Boroujeni A, et al. Clinicopathologic and genomic analysis of TP53-Mutated endometrial carcinomas. Clin Cancer Res. 2021;27(9):2613–23. 33602681 10.1158/1078-0432.CCR-20-4436 PMC8530276

