# Supplementary figures and images for: Survival outcome prediction of esophageal squamous cell carcinoma patients based on radiomics and mutation signature
Source: Cancer Imaging. 2025 Jan 31;25:9. doi: 10.1186/s40644-024-00821-5 (PMC11783911; doi:10.1186/s40644-024-00821-5)

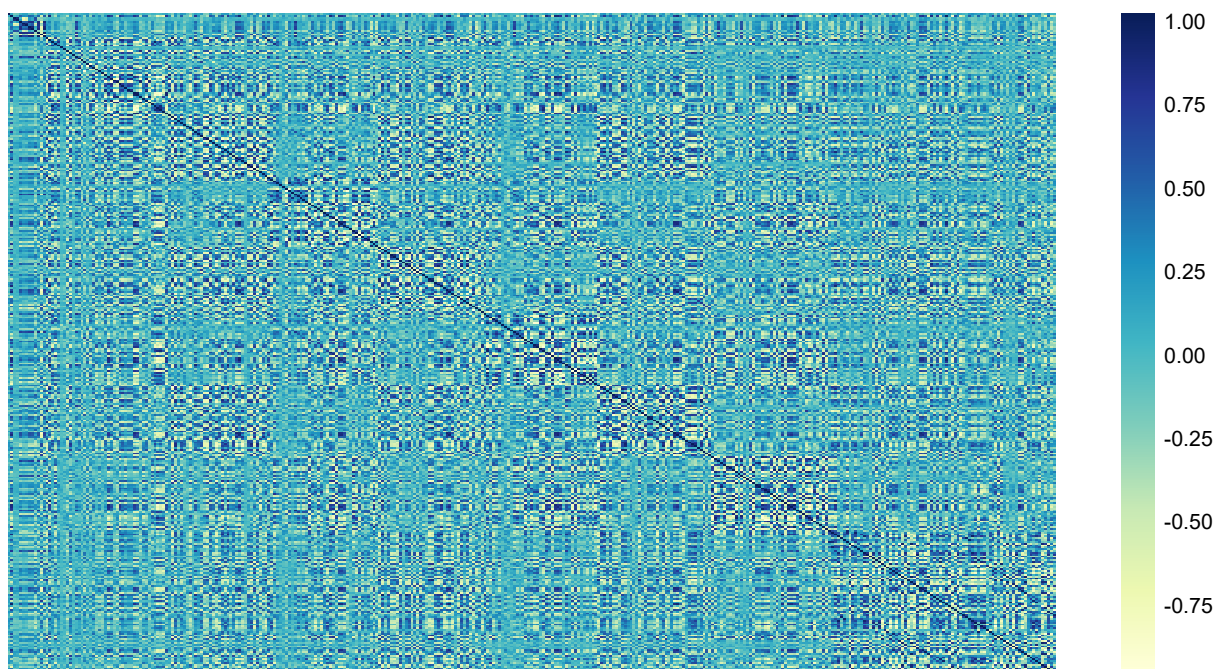

(a)

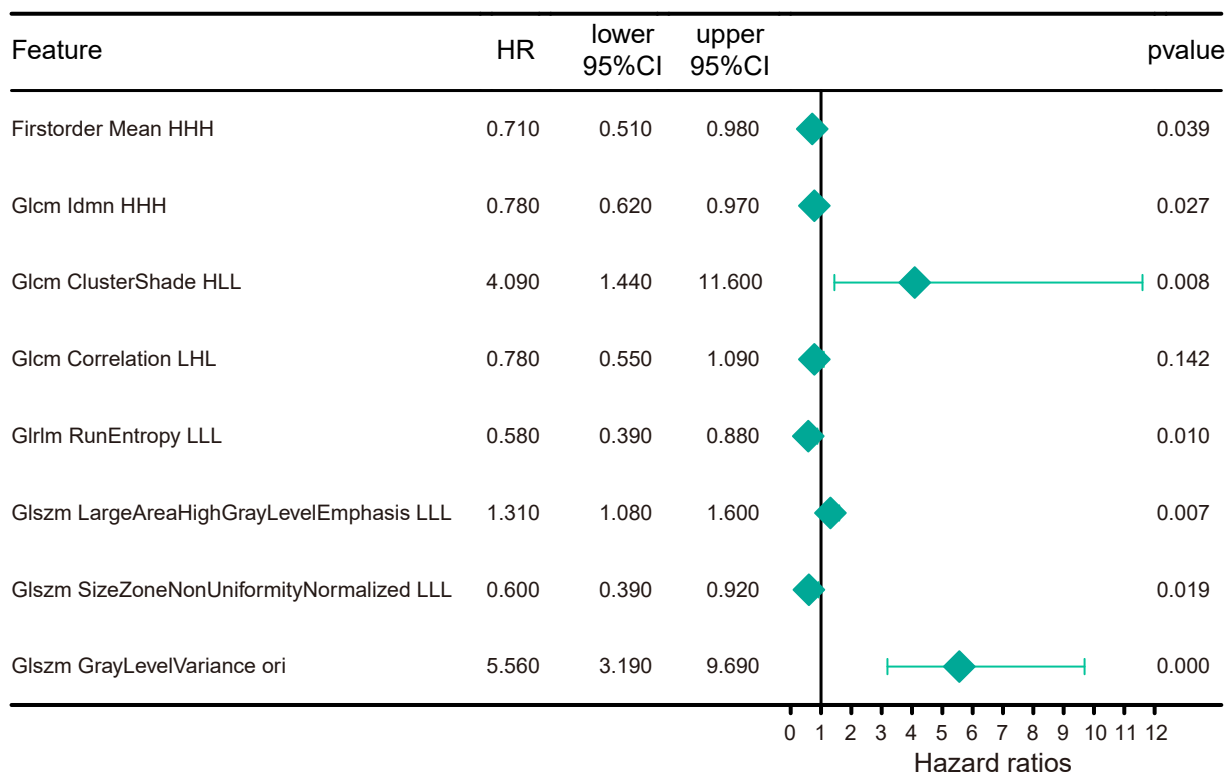

(b)

Supplement: Supplementary file 1 — Supplementary Material 1. Fig. S1 (a) Spearman correlation between 359 features. The pairwise correlation was less than 0.95. (b) Forest plot of multivariate Cox regression analysis. [file 40644_2024_821_MOESM1_ESM.pdf]

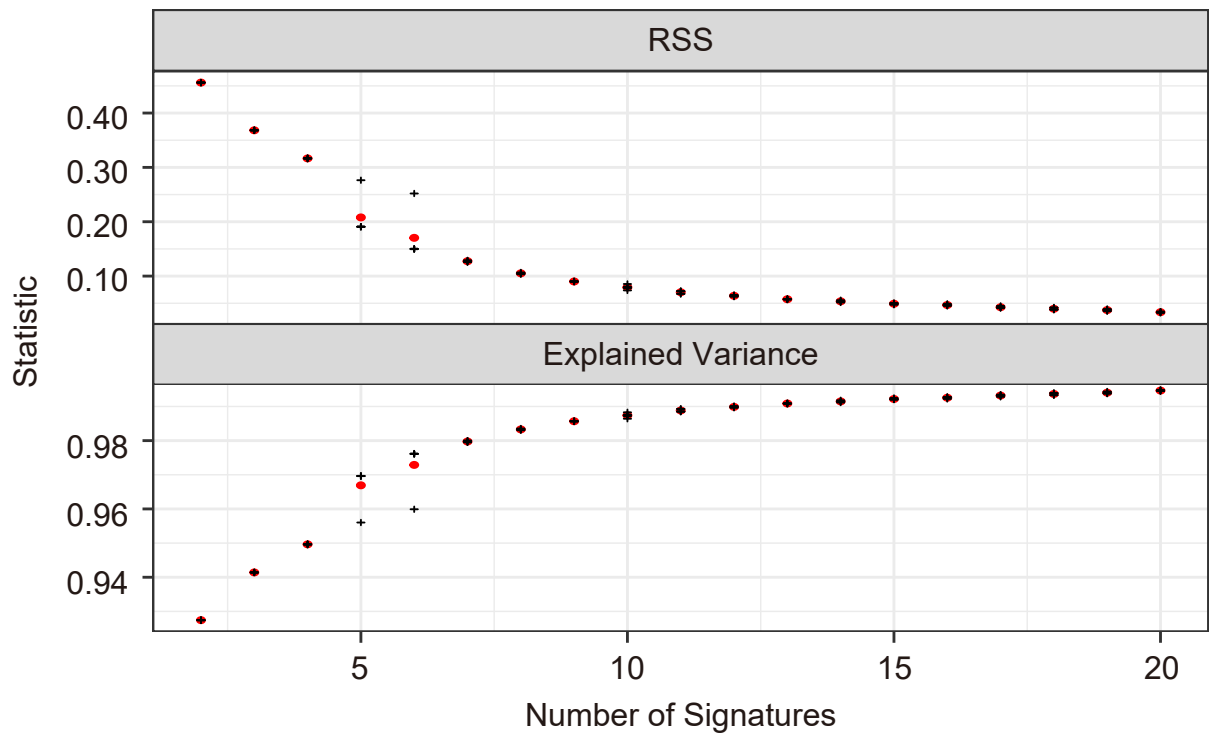

(a)

205-WGS ESCCs cohort

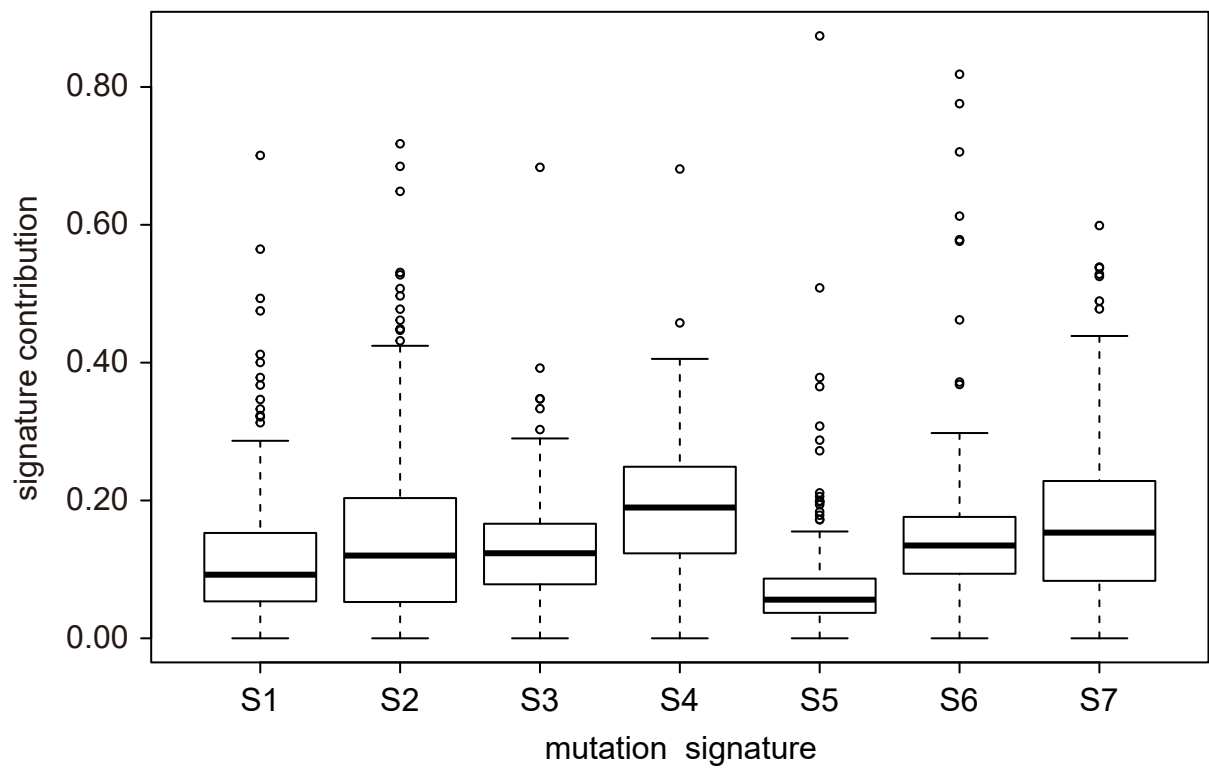

(b)

Supplement: Supplementary file 2 — Supplementary Material 2. Fig. S2 (a) Upper, residual sum of squares (RSS) of different signature number selections. Lower, percentage of variance explained by the selection of different signature numbers. (b) Relative contributions of the seven mutational features. [file 40644_2024_821_MOESM2_ESM.pdf]

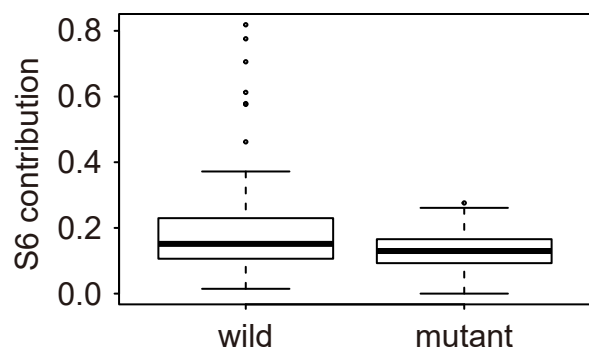

TP53

(a)

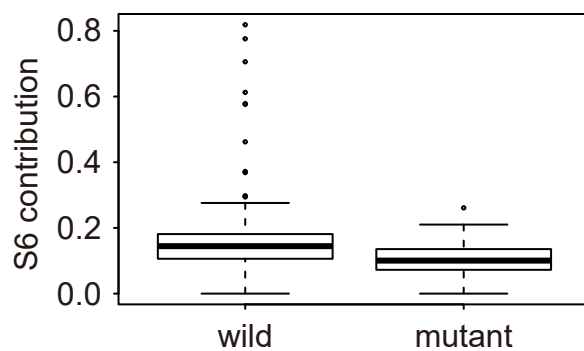

MUC16

(b)

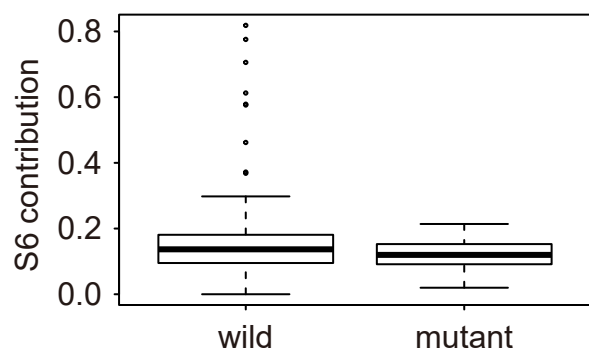

FAT1

(c)

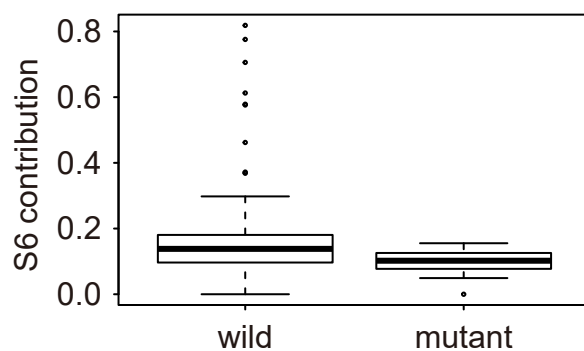

LRP1B

(d)

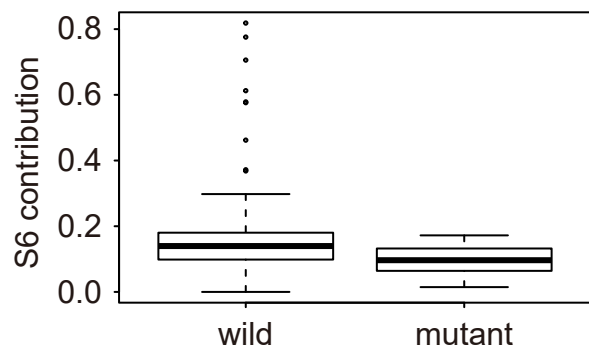

SI

(e)

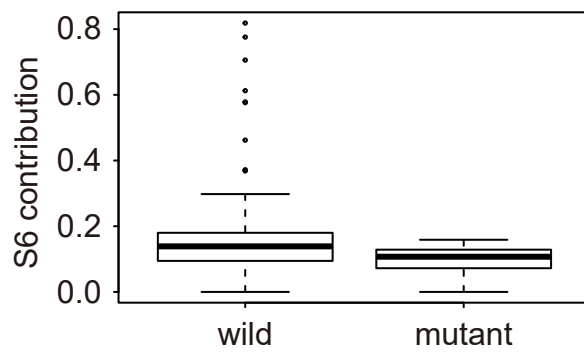

USH2A

(f)

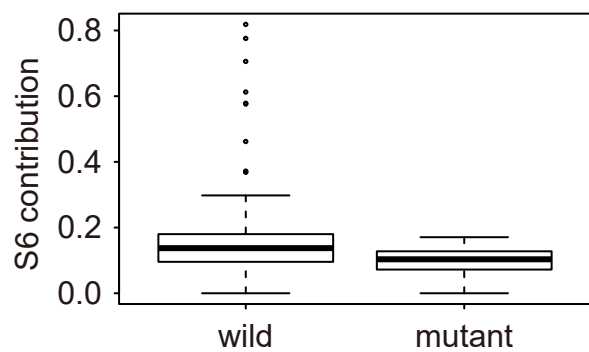

DMD

(g)

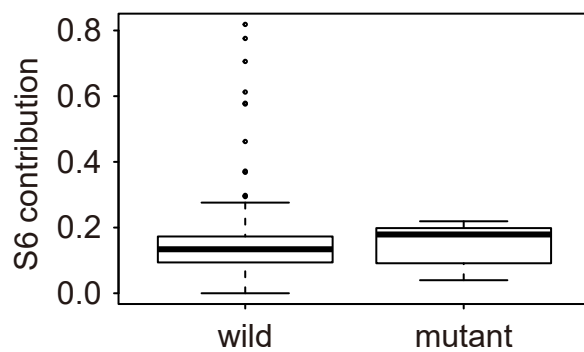

MDN1

(h)

Supplement: Supplementary file 3 — Supplementary Material 3. Fig. S3 The proportion of S6 positive patients with or without these eight mutated genes. [file 40644_2024_821_MOESM3_ESM.pdf]
